# Intimate partner violence and firearm purchasing: cross-sectional analysis of statewide survey data from California and Louisiana adults

**DOI:** 10.1186/s12889-025-25361-w

**Published:** 2025-11-25

**Authors:** Jakana Thomas, Nicole E Johns, Annika Li, Gennifer Kully, Anita Raj

**Affiliations:** 1 School of Global Policy and Strategy and Department of Political Science, University of California San Diego, San Diego, CA USA; 2 Center on Gender Equity and Health, University of California San Diego, San Diego, CA USA; 3 Department of Education Studies, University of California San Diego, San Diego, CA USA; 4 Newcomb Institute and Celia Scott Weatherhead School of Public Health and Tropical Medicine, Tulane University, New Orleans, Louisiana USA

**Keywords:** Intimate partner violence, Firearms, Gun ownership

## Abstract

**Objective:**

To assess associations between recent intimate partner violence (IPV) (victimization and perpetration) on firearm ownership and recent firearm purchase.

**Methods:**

Analysis of statewide online surveys on violence conducted in 2023 in California and Louisiana, respectively. We examine whether past year IPV was associated with firearm ownership using regression models adjusting for sociodemographic covariates and mental health. Survey participants were adults from California (*N* = 3,560), with data collected between March and May 2023 and Louisiana (*N* = 1,081), collected between May and June 2023.

**Results:**

In fully adjusted models, IPV victimization was associated with three times higher odds of current firearm ownership (AOR 2.93, 95% CI 1.80–4.77, *p* < 0.001), and IPV perpetration was associated with 10 times higher odds of ownership (AOR 9.85, 95% CI 3.95–25.59, *p* < 0.001). IPV victimization was associated with three times higher odds of past year firearm purchase (AOR 3.15, 95% CI 1.90–5.22, *p* < 0.001), and perpetration was associated with five times higher odds of purchase (AOR 4.98, 95% CI 2.32–10.65, *p* < 0.001).

**Conclusions:**

Findings indicate those experiencing and perpetrating IPV are significantly more likely to report firearm ownership and recent purchase of a firearm.

**Supplementary Information:**

The online version contains supplementary material available at 10.1186/s12889-025-25361-w.

## Background

Firearm ownership among men in the United States has stabilized while it continues to increase for women [1], with both women and men reporting desire for firearms for protection [2]. Women who have been victimized by an intimate partner with a firearm are significantly more likely to engage in self-protective behaviors that involve firearms, including purchasing or borrowing a firearm, than women without those experiences [3, 4]. Women who have taken such protective steps are also more likely to believe that firearm ownership will protect them from future intimate partner violence (IPV) [3, 4] and may even have been encouraged by others to acquire firearms to protect themselves [5]. They are also more likely to obtain a firearm for protection if their abuser has threatened them with a firearm previously. Less is understood about these issues for men and gender expansive people (i.e., non-binary or other gender non-conforming people).

Firearm-related IPV, or incidents where a firearm is used to intimidate an intimate partner, is well documented [6–8], with firearm threats made not only against the victim [8] but also against their pets and other loved ones [9]. During breakups, firearms have been used to reassert control over a spouse, even in cases with no prior history of abuse [10]. Firearm-related IPV substantially increases risk for intimate partner homicide (IPH) for women [11–13], and evidence indicates that IPH is often preceded by multiple firearm-related IPV incidents [12]. Most IPH in the U.S. is due to a firearm [11, 14, 15], with highest risk seen for Black women affected by IPV [16, 17]. Understanding and addressing firearm-related IPV can reduce the risk of IPH.

Fire-arm related IPV has largely been studied with a focus on women victims. Studies find that firearm-related IPV victimization is reported across all genders [18, 19] but is more likely for women than men [6, 9]. Older data suggest that women’s use of firearms to protect themselves from an abuser is rare, reported by only 5% [20], but this may be shifting in light of the increasing firearm ownership reported by women, and as women may view firearms as a means of protection against an abusive partner. Importantly, more recent evidence indicates that women are twice as likely to use a firearm to perpetrate IPH when their partner had previously wielded a weapon against them [10].

While the literature highlights that firearm purchases are increasing and the presence of firearms in the context of IPV-affected relationships can increase risk for IPH, we do not have clear evidence on whether recent exposure to IPV is in fact associated with higher odds of a firearm purchase. This study seeks to address this gap in the literature using data from statewide samples of adults in California and Louisiana, the former state with among the most restrictive firearm polices in the country and the latter with among the laxest firearm policies. Using data from these two different contexts enables us to include Americans from vastly different backgrounds, and with different firearm-related norms and legislative contexts around IPV and firearm ownership. The latter is particularly important as research shows significant reductions in IPH where more stringent laws restrict domestic violence offenders from possessing firearms [21–23]. These findings can guide firearm policy considerations to affect IPV and IPH.

## Methods

We utilized cross-sectional data from the 2023 Violence Experiences (VEX) surveys conducted in California and Louisiana, respectively [18, 19]. These surveys assessed experiences of different forms of violence across the lifespan, as well as demographics and attitudinal variables. Both surveys are state-representative online surveys of adults age 18 + that were implemented by the survey firm NORC at the University of Chicago in Spring 2023, using their probability-based and non-probability based online panels. The 2023 California Violence Experiences survey (CalVEX) surveyed 3,560 Californian residents between March and May 2023, and the 2023 Louisiana Violence Experiences (LaVEX) polled 1,081 Louisiana residents between May and June 2023. The participation rates were 21% for the CalVEX survey and 27% for the LaVEX survey, which is within an acceptable range for online surveys to yield population-based estimates. For further details on sampling and participation, please see the CalVEX and LaVEX 2023 reports [18, 19]. The online survey took approximately 15 min to complete, and participants were compensated with the cash equivalent of approximately $4.00.

### Measures

We included two outcomes of interest, firearm ownership and past year firearm purchase, using a single item from our survey which asked, “Have you obtained a firearm in the past 12 months?”, with response options “1. Yes, I have obtained a firearm for the first time”, “2. Yes, this is not my first firearm”, “3. No, but I have a firearm” and “4. No, and I do not have a firearm at this time.” *Firearm ownership* is indicated if the respondent selected any of the first three options. *Firearm purchase* is indicated if the respondent indicated either of the first two options. While obtaining a firearm in the past year could technically have included receiving the firearm as a gift or stealing the firearm, we refer to this as ‘firearm purchase’ throughout.

Our primary predictors of interest were past year IPV victimization and past year IPV perpetration. The VEX surveys ask respondents about 22 forms of “physical, sexual, and emotional violence from a current or romantic or sexual partner. This can include a spouse, someone you were casually dating, boyfriends, girlfriends, or sexual partners.” This violence and mistreatment can include a range of unwanted behaviors, including physical and sexual violence, insults, harassment, restrictions on other relationships, and violent threats against the respondent or loved ones. IPV victimization is indicated if a respondent reported experiencing any of the 22 listed forms of IPV within the past year. IPV perpetration was assessed for only 9 forms of physical or sexual violence, and it was indicated if the respondent reported committing any of those forms of violence against a current or former romantic or sexual partner within the past year.

Our covariates included sociodemographic variables selected a priori based on established associations with either IPV experience or firearm purchase or ownership. These included: gender (woman, man, non-binary/genderqueer/gender fluid/other self-described gender identity), political ideology (very liberal, somewhat liberal, moderate, somewhat conservative, very conservative), education level (less than high school degree, completed high school/some college/associate’s degree, bachelor’s degree/four year college degree, graduate degree), race/ethnicity (non-Hispanic white, non-Hispanic Black, non-Hispanic Asian, Hispanic, Other/multiple races), annual income (<$30,000, $30,000-<$60,000, $60,000-<$100,000, $100,000+), age (continuous years), current marital status (yes, no), identifies with a specific religion (yes, no), and state (California or Louisiana). We also account for an individual’s reported past 2-week depression and anxiety (mental health) symptoms using the PHQ-4 measure (Kroenke et al., 2009), which scores respondents as experiencing normal, mild, moderate, or severe symptoms [24].

### Data analysis

We present descriptive statistics for the study population overall with regards to firearm ownership and purchase, past year IPV experiences, and key sociodemographic indicators. We then present bivariate comparisons of past year IPV experiences and sociodemographic factors by firearm ownership and by firearm purchase, testing significant associations via Pearson’s chi-squared statistics for categorical measures and adjusted Wald tests for the sole continuous measure, age. We then conduct a series of logistic regression models to examine the association between experiences of IPV and each firearm measure. We conduct the following five models first for firearm ownership, and then for past year firearm purchase:

- Model 1. Unadjusted model including only past year IPV victimization.
- Model 2. Unadjusted model including only past year IPV perpetration.
- Model 3. Adjusted model including past year IPV victimization and all sociodemographic characteristics.
- Model 4. Adjusted model including past year IPV perpetration and all sociodemographic characteristics.
- Model 5. Adjusted model including past year IPV victimization, past year IPV perpetration, and all sociodemographic characteristics.

We also conduct several sensitivity analyses. First, we examine whether past year IPV experiences are associated with first-time firearm purchase specifically, limiting the sample to individuals who procured a weapon in the past year and examining likelihood of first-time purchase versus purchase among those who already own firearms, replicating Models 1–5 above. Second, we stratify Model 5 by state to examine whether overall associations differ meaningfully between California and Louisiana. Finally, we stratify Model 5 by respondent gender, to assess whether gender impacts the relationships between IPV and firearm ownership and purchasing. Due to the relatively small number of non-binary gender respondents (*n* = 77), only women and men are examined in this sensitivity analysis.

All analyses took provided survey weights into account. Statistical significance was set at *p* < 0.05 for all comparisons and 95% confidence intervals (CIs) are reported for adjusted odds ratios (AORs) throughout.

### Ethical review

All participants provided consent to NORC for inclusion in the survey panel at the time of enrollment, and they could opt out of the panel at any time. Participants are also able to opt out of the VEX surveys entirely or at any time during survey participation. All study procedures were reviewed and approved by the relevant institutional review boards.

## Results

The CalVEX and LaVEX 2023 surveys had 4,641 total respondents. Of these, *n* = 30 (0.6%) were missing responses to the firearm ownership and purchase survey item and were thus excluded from analyses, for a final analytic sample of *n* = 4,611.

A quarter of all respondents reported current firearm ownership (26.1%) and 6.2% reported purchasing a firearm within the past year (see Table 1). Men and more conservative, white, higher income, older, currently married, and religious respondents were more likely to report current firearm ownership than their counterparts in bivariate comparisons. Louisianans were about twice as likely to report firearm ownership as Californians (41.9% vs. 21.3%, *p* < 0.001). Men, younger and religious respondents, as well as respondents who indicated moderate past two-week mental health symptoms, were more likely to report past-year firearm purchase than their counterparts, and Louisianans were again twice as likely to report past-year firearm purchase than Californians (10.1% vs. 5.0%, *p* < 0.001).

**Table 1:** Sociodemographics & predictors of interest, overall and by firearm ownership and purchase status

	No. (%)						
	Total (*n* = 4611)	Current firearm ownership			Past-year firearm purchase		
Variable		No (*n* = 3399)	Yes (*n* = 1212)	*P* value	No (*n* = 4255)	Yes (*n* = 356)	*P* value
Gender				< 0.001			< 0.001
Woman	2740 (50.6)	2152 (80.1)	588 (19.9)		2591 (96.5)	149 (3.5)	
Man	1794 (47.8)	1199 (67.6)	595 (32.4)		1594 (90.8)	200 (9.2)	
Non-binary	77 (1.6)	48 (64.6)	29 (35.4)		70 (96.5)	7 (3.5)	
Ideology				< 0.001			0.02
Very liberal	625 (14.0)	496 (83.3)	129 (16.7)		565 (93.9)	60 (6.1)	
Somewhat liberal	673 (15.1)	561 (86.1)	112 (13.9)		644 (97.1)	29 (2.9)	
Moderate	2215 (46.1)	1631 (72.7)	583 (27.3)		2055 (93.8)	160 (6.2)	
Somewhat conservative	635 (14.4)	408 (63.3)	227 (36.7)		575 (91.9)	60 (8.2)	
Very conservative	400 (10.4)	245 (60.1)	155 (39.9)		355 (91.1)	45 (8.9)	
Education				0.14			0.39
Less than HS degree	277 (11.0)	209 (79.5)	68 (20.5)		250 (91.5)	27 (8.6)	
Completed HS/Some college/AD	2504 (53.3)	1835 (72.1)	669 (27.9)		2302 (93.8)	202 (6.2)	
BA/4 year college degree	1058 (20.2)	784 (75.0)	274 (25.0)		987 (94.7)	71 (5.3)	
Graduate degree	772 (15.5)	571 (74.4)	201 (25.6)		716 (94.2)	56 (5.8)	
Race/ethnicity				< 0.001			0.07
Non-Hispanic White	1779 (42.5)	1168 (65.0)	611 (35.0)		1655 (94.4)	124 (5.6)	
Non-Hispanic Black	1050 (10.8)	812 (74.2)	238 (25.8)		955 (90.2)	95 (9.8)	
Non-Hispanic Asian	643 (13.00)	540 (85.0)	103 (15.0)		608 (95.7)	35 (4.3)	
Hispanic	900 (29.7)	706 (82.1)	194 (18.0)		818 (93.5)	82 (6.5)	
Other/multiple races	239 (4.1)	173 (70.3)	66 (29.7)		219 (92.6)	20 (7.4)	
Income				< 0.001			0.84
<$30,000	1135 (25.3)	1055 (79.6)	280 (20.4)		1227 (93.7)	108 (6.3)	
$30,000-<$60,000	1080 (22.3)	819 (75.8)	261 (24.2)		999 (94.5)	81 (5.5)	
$60,000-<$100,000	945 (21.6)	654 (69.8)	291 (30.2)		873 (94.0)	72 (6.0)	
$100,000+	1251 (30.7)	871 (70.6)	380 (29.4)		1156 (93.2)	95 (6.8)	
Age				< 0.001			< 0.001
Mean age (SD), years	47.7 (17.4)	46.6 (17.1)	50.9 (17.9)		48.2 (17.3)	40.1 (16.8)	
Mental health symptom score				0.09			< 0.001
Normal	2245 (55.5)	1663 (72.6)	582 (27.4)		2139 (95.3)	106 (4.7)	
Mild	1151 (25.4)	862 (75.4)	289 (24.6)		1056 (93.2)	95 (6.8)	
Moderate	606 (11.7)	416 ((71.7)	190 (28.3)		513 (88.9)	93 (11.1)	
Severe	457 (7.5)	354 (80.9)	103 (19.1)		416 (92.2)	41 (7.8)	
Currently married				< 0.001			0.22
No	2751 (55.5)	2170 (78.7)	581 (21.3)		2559 (94.4)	192 (5.7)	
Yes	1850 (44.5)	1221 (67.9)	629 (32.2)		1687 (93.1)	163 (6.9)	
Religious				< 0.001			< 0.001
No	1300 (29.5)	1027 (80.0)	273 (20.0)		1235 (96.4)	65 (3.6)	
Yes	3273 (70.6)	2338 (71.2)	935 (28.8)		2984 (92.7)	289 (7.3)	
State				< 0.001			< 0.001
CA	3535 (76.6)	2746 (78.7)	789 (21.3)		3320 (95.0)	215 (5.0)	
LA	1076 (23.4)	653 (58.1)	423 (41.9)		935 (89.9)	141 (10.1)	
IPV victimization in past year				< 0.001			< 0.001
No	4213 (93.9)	3173 (75.2)	1040 (24.9)		3958 (94.9)	255 (5.1)	
Yes	351 (6.1)	193 (53.3)	158 (46.7)		252 (76.0)	99 (24.0)	
IPV perpetration in past year				< 0.001			< 0.001
No	4517 (98.7)	3371 (74.6)	1146 (25.4)		4209 (94.3)	308 (5.7)	
Yes	94 (1.3)	28 (18.7)	66 (81.3)		46 (54.8)	48 (45.2)	

One in 16 respondents (6.1%) reported IPV victimization in the past year, and 1.3% reported perpetration of IPV in that time frame. In bivariate tests, firearm ownership was reported significantly more frequently by those who reported past year IPV victimization (46.7% vs. 24.9%, *p* < 0.001) or perpetration (81.3% vs. 25.4%, *p* < 0.001), compared to those who did not have such IPV experiences. Past year firearm purchase was also significantly more common among those who reported past year IPV victimization (24.0% vs. 5.1%, *p* < 0.001) or perpetration (45.2% vs. 5.7%, *p* < 0.001).

Past year IPV victimization and perpetration were each associated with greater likelihood of firearm ownership in both unadjusted and adjusted logistic regression models (see Table 2). In the fully adjusted model accounting for both indicators of IPV experiences, IPV victimization was associated with three times higher odds of current firearm ownership (Model 5 AOR 2.93, 95% CI 1.80–4.77, *p* < 0.001), and IPV perpetration was associated with 10 times higher odds of ownership (Model 5 AOR 9.85, 95% CI 3.95–25.59, *p* < 0.001). Male gender, conservative ideology, white race, higher income, older age, current marriage, normal mental health symptomology, and Louisiana residence were all also significantly associated with greater likelihood of current firearm ownership in fully adjusted models.

**Table 2:** Current firearm ownership

	Model 1			Model 2			Model 3			Model 4			Model 5		
	OR	95% CI	*p*-value	OR	95% CI	*p*-value	AOR	95% CI	*p*-value	AOR	95% CI	*p*-value	AOR	95% CI	*p*-value
IPV victimization in past year															
No (reference)															
Yes	2.65	1.85,3.81	<0.0001				3.89	2.45,6.19	<0.0001				2.93	1.80,4.77	<0.0001
IPV perpetration in past year															
No (reference)															
Yes				12.78	5.87,27.82	<0.0001				18.46	7.50,45.42	<0.0001	9.85	3.95,24.59	<0.0001
Gender															
Woman (reference)															
Man							1.97	1.57,2.46	<0.0001	2.00	1.60,2.49	<0.0001	1.97	1.57,2.46	<0.0001
Non-binary							3.95	1.31,11.94	0.0148	4.42	1.36,14.34	0.0133	4.12	1.31,13.00	0.0156
Ideology															
Very liberal (reference)															
Somewhat liberal							0.95	0.60,1.52	0.8403	0.96	0.59,1.54	0.8574	0.99	0.61,1.59	0.9528
Moderate							1.91	1.33,2.73	0.0004	1.90	1.31,2.76	0.0007	1.94	1.34,2.82	0.0004
Somewhat conservative							2.36	1.56,3.58	0.0001	2.43	1.59,3.71	<0.0001	2.45	1.60,3.76	<0.0001
Very conservative							2.58	1.59,4.17	0.0001	2.58	1.57,4.25	0.0002	2.66	1.62,4.37	0.0001
Education															
Less than HS degree (reference)															
Completed HS/Some college/AD							1.07	0.68,1.69	0.7752	1.11	0.70,1.74	0.6639	1.12	0.71,1.76	0.6381
BA/4 year college degree							0.82	0.50,1.35	0.4367	0.83	0.51,1.37	0.472	0.86	0.52,1.41	0.5408
Graduate degree							0.77	0.46,1.31	0.3397	0.80	0.47,1.35	0.4035	0.80	0.47,1.36	0.4137
Race/ethnicity															
Non-Hispanic White (reference)															
Non-Hispanic Black							0.65	0.46,0.91	0.0126	0.64	0.45,0.89	0.0089	0.63	0.45,0.89	0.0092
Non-Hispanic Asian							0.42	0.30,0.60	<0.0001	0.42	0.30,0.60	<0.0001	0.43	0.30,0.61	<0.0001
Hispanic							0.55	0.39,0.76	0.0004	0.56	0.40,0.78	0.0006	0.54	0.39,0.76	0.0005
Other/multiple races							1.13	0.71,1.81	0.6034	1.15	0.70,1.87	0.5867	1.15	0.71,1.86	0.5688
Income															
<$30,000 (reference)															
$30,000-<$60,000							1.31	0.93,1.86	0.1271	1.30	0.92,1.85	0.14	1.31	0.92,1.85	0.1363
$60,000-<$100,000							1.72	1.22,2.44	0.0021	1.73	1.23,2.44	0.0017	1.74	1.22,2.46	0.0020
$100,000+							1.94	1.35,2.80	0.0004	1.91	1.33,2.75	0.0005	1.95	1.35,2.82	0.0004
Age															
*Years*							1.01	1.00,1.01	0.0634	1.01	1.00,1.01	0.1302	1.01	1.00,1.01	0.0472
Mental health symptom score															
Normal (reference)															
Mild							0.92	0.70,1.20	0.5308	0.91	0.70,1.20	0.5117	0.89	0.68,1.16	0.3822
Moderate							1.39	0.98,1.98	0.0684	1.43	1.00,2.02	0.0479	1.36	0.95,1.95	0.0938
Severe							0.62	0.41,0.95	0.0278	0.68	0.44,1.04	0.076	0.59	0.38,0.92	0.0191
Currently married															
No (reference)															
Yes							1.45	1.15,1.84	0.0018	1.47	1.16,1.87	0.0014	1.46	1.15,1.85	0.0019
Religious															
No (reference)															
Yes							1.18	0.91,1.53	0.2054	1.24	0.96,1.61	0.0953	1.17	0.90,1.52	0.2357
State															
CA (reference)															
LA							2.45	1.87,3.20	<0.0001	2.35	1.80,3.07	<0.0001	2.45	1.87,3.22	<0.0001

Past year IPV victimization and perpetration were also each associated with greater likelihood of past year firearm purchase in both unadjusted and adjusted logistic regression models (see Table 3). In the fully adjusted model accounting for both indicators of IPV experiences, IPV victimization was associated with three times higher odds of past year firearm purchase (Model 5 AOR 3.15, 95% CI 1.90–5.22, *p* < 0.001), and IPV perpetration was associated with five times higher odds of purchase (Model 5 AOR 4.98, 95% CI 2.32–10.65, *p* < 0.001). Male gender, younger age, current marriage, moderate mental health symptomology, religious affiliation, and Louisiana residence were all also significantly associated with greater likelihood of past year firearm purchase in fully adjusted models.

**Table 3:** Past year firearm purchase

	Model 1			Model 2			Model 3			Model 4			Model 5		
	OR	95% CI	*p*-value	OR	95% CI	*p*-value	AOR	95% CI	*p*-value	AOR	95% CI	*p*-value	AOR	95% CI	*p*-value
IPV victimization in past year															
No (reference)															
Yes	5.84	3.86,8.83	<0.0001				4.09	2.61,6.40	<0.0001				3.15	1.90,5.22	<0.0001
IPV perpetration in past year															
No (reference)															
Yes				13.64	6.97,26.70	<0.0001				9.42	4.79,18.53	<0.0001	4.98	2.32,10.65	<0.0001
Gender															
Woman (reference)															
Man							3.07	2.09,4.52	<0.0001	3.14	2.13,4.63	<0.0001	3.12	2.10,4.63	<0.0001
Non-binary							0.29	0.04,2.13	0.2245	0.41	0.08,2.13	0.2921	0.31	0.05,2.00	0.2185
Ideology															
Very liberal (reference)															
Somewhat liberal							0.58	0.28,1.20	0.143	0.60	0.28,1.27	0.1778	0.64	0.31,1.32	0.2256
Moderate							1.02	0.64,1.64	0.9293	1.04	0.62,1.75	0.8728	1.06	0.64,1.75	0.8145
Somewhat conservative							1.46	0.81,2.66	0.2114	1.59	0.85,2.98	0.1482	1.61	0.87,2.97	0.1299
Very conservative							1.64	0.79,3.40	0.1833	1.67	0.77,3.61	0.1916	1.73	0.81,3.70	0.1561
Education															
Less than HS degree (reference)															
Completed HS/Some college/AD							0.95	0.51,1.79	0.8803	0.99	0.52,1.90	0.9852	0.99	0.51,1.91	0.9747
BA/4 year college degree							0.81	0.40,1.63	0.5492	0.84	0.40,1.74	0.6362	0.83	0.40,1.72	0.6131
Graduate degree							0.92	0.43,1.94	0.8199	0.96	0.44,2.08	0.917	0.93	0.43,2.02	0.8636
Race/ethnicity															
Non-Hispanic White (reference)															
Non-Hispanic Black							1.44	0.86,2.42	0.1643	1.40	0.84,2.35	0.1964	1.42	0.84,2.40	0.194
Non-Hispanic Asian							0.82	0.43,1.59	0.5587	0.82	0.43,1.58	0.5573	0.85	0.44,1.63	0.6195
Hispanic							1.08	0.60,1.93	0.7965	1.08	0.61,1.92	0.7898	1.09	0.61,1.96	0.7758
Other/multiple races							1.38	0.61,3.16	0.4411	1.46	0.59,3.60	0.4168	1.40	0.58,3.35	0.4526
Income															
<$30,000 (reference)															
$30,000-<$60,000							1.08	0.64,1.80	0.7813	1.11	0.65,1.91	0.6996	1.08	0.63,1.85	0.7740
$60,000-<$100,000							1.32	0.76,2.29	0.3317	1.32	0.76,2.29	0.3191	1.33	0.76,2.35	0.3195
$100,000+							1.46	0.80,2.67	0.2192	1.46	0.78,2.73	0.232	1.48	0.80,2.74	0.2164
Age															
*Years*							0.97	0.95,0.98	<0.0001	0.96	0.95,0.98	<0.0001	0.97	0.95,0.98	<0.0001
Mental health symptom score															
Normal (reference)															
Mild							1.31	0.79,2.20	0.2956	1.37	0.82,2.30	0.2328	1.25	0.74,2.12	0.4058
Moderate							1.94	1.15,3.26	0.0131	2.04	1.22,3.41	0.0068	1.86	1.10,3.15	0.0205
Severe							1.27	0.67,2.40	0.468	1.43	0.75,2.74	0.2752	1.19	0.61,2.34	0.6083
Currently married															
No (reference)															
Yes							1.61	1.08,2.38	0.0181	1.63	1.08,2.46	0.0204	1.60	1.07,2.40	0.0233
Religious															
No (reference)															
Yes							1.89	1.19,2.99	0.007	1.99	1.25,3.16	0.0037	1.86	1.17,2.96	0.0091
State															
CA (reference)															
LA							2.03	1.34,3.08	0.0008	1.94	1.29,2.92	0.0015	2.01	1.32,3.06	0.0011

Finally, Table 4 replicates Model 5 for each firearm outcome by respondent gender. Significant positive associations between past year IPV experiences and firearm ownership and purchase remain present at comparable magnitudes for both examined genders.

**Table 4:** Fully adjusted regression models [Model 5] of firearm ownership and purchase, stratified by gender

	FIREARM OWNERSHIP									FIREARM PURCHASE								
	Combined sample			Women			Men			Combined sample			Women			Men		
	AOR	95% CI	*p*-value	AOR	95% CI	*p*-value	AOR	95% CI	*p*-value	AOR	95% CI	*p*-value	AOR	95% CI	*p*-value	AOR	95% CI	*p*-value
IPV victimization in past year																		
No (reference)																		
Yes	2.93	1.80,4.77	<0.0001	2.57	1.17,5.67	0.0192	3.06	1.58,5.96	0.001	3.15	1.90,5.22	<0.0001	2.53	1.17,5.50	0.0187	3.65	1.98,6.72	<0.0001
IPV perpetration in past year																		
No (reference)																		
Yes	9.85	3.95,24.59	<0.0001	21.02	4.79,92.28	0.0001	5.96	1.82,19.46	0.0031	4.98	2.32,10.65	<0.0001	7.97	2.48,25.61	0.0005	6.04	2.17,16.82	0.0006
Gender																		
Woman (reference)																		
Man	1.97	1.57,2.46	<0.0001							3.12	2.10,4.63	<0.0001						
Non-binary	4.12	1.31,13.00	0.0156							0.31	0.05,2.00	0.2185						
Ideology																		
Very liberal (reference)																		
Somewhat liberal	0.99	0.61,1.59	0.9528	0.99	0.53,1.88	0.9855	0.98	0.49,1.94	0.9469	0.64	0.31,1.32	0.2256	0.61	0.22,1.70	0.3441	0.66	0.25,1.76	0.4073
Moderate	1.94	1.34,2.82	0.0004	2.48	1.46,4.19	0.0007	1.69	1.05,2.72	0.0311	1.06	0.64,1.75	0.8145	1.17	0.47,2.93	0.7331	1.07	0.58,1.99	0.8205
Somewhat conservative	2.45	1.60,3.76	<0.0001	2.64	1.44,4.86	0.0017	2.39	1.35,4.21	0.0027	1.61	0.87,2.97	0.1299	1.07	0.38,3.03	0.8991	1.99	0.92,4.33	0.0825
Very conservative	2.66	1.62,4.37	0.0001	3.04	1.52,6.05	0.0016	2.36	1.20,4.64	0.0124	1.73	0.81,3.70	0.1561	2.58	0.78,8.47	0.1188	1.54	0.58,4.09	0.391
Education																		
Less than HS degree (reference)																		
Completed HS/Some college/AD	1.12	0.71,1.76	0.6381	1.61	0.83,3.11	0.1583	1.26	0.68,2.34	0.4661	0.99	0.51,1.91	0.9747	5.73	1.55,21.15	0.0087	0.69	0.33,1.48	0.3441
BA/4 year college degree	0.86	0.52,1.41	0.5408	1.10	0.54,2.25	0.7859	1.02	0.52,2.00	0.9614	0.83	0.40,1.72	0.6131	7.00	1.59,30.88	0.0102	0.48	0.20,1.13	0.091
Graduate degree	0.80	0.47,1.36	0.4137	1.50	0.68,3.30	0.3148	0.76	0.37,1.53	0.4346	0.93	0.43,2.02	0.8636	5.73	1.16,28.34	0.0323	0.60	0.24,1.48	0.2655
Race/ethnicity																		
Non-Hispanic White (reference)																		
Non-Hispanic Black	0.63	0.45,0.89	0.0092	0.63	0.39,1.02	0.0607	0.68	0.42,1.11	0.1221	1.42	0.84,2.40	0.194	2.18	0.90,5.26	0.0843	0.99	0.55,1.78	0.9809
Non-Hispanic Asian	0.43	0.30,0.61	<0.0001	0.32	0.17,0.61	0.0005	0.52	0.34,0.81	0.0038	0.85	0.44,1.63	0.6195	1.74	0.52,5.81	0.3651	0.66	0.31,1.43	0.2953
Hispanic	0.54	0.39,0.76	0.0005	0.39	0.23,0.66	0.0006	0.68	0.43,1.08	0.1006	1.09	0.61,1.96	0.7758	1.63	0.62,4.31	0.3233	0.95	0.46,1.98	0.8934
Other/multiple races	1.15	0.71,1.86	0.5688	0.70	0.36,1.38	0.3040	2.09	1.04,4.22	0.0388	1.40	0.58,3.35	0.4526	1.44	0.39,5.37	0.5886	1.39	0.41,4.71	0.5931
Income																		
<$30,000 (reference)																		
$30,000-<$60,000	1.31	0.92,1.85	0.1363	1.57	0.96,2.57	0.0753	1.14	0.68,1.92	0.6171	1.08	0.63,1.85	0.7740	0.61	0.25,1.45	0.2626	1.46	0.73,2.91	0.2822
$60,000-<$100,000	1.74	1.22,2.46	0.0020	2.08	1.28,3.37	0.0030	1.62	0.96,2.72	0.0682	1.33	0.76,2.35	0.3195	0.91	0.39,2.12	0.8291	1.68	0.76,3.71	0.2004
$100,000+	1.95	1.35,2.82	0.0004	2.15	1.26,3.65	0.0049	1.95	1.15,3.32	0.0136	1.48	0.80,2.74	0.2164	1.04	0.43,2.51	0.9280	1.89	0.80,4.43	0.1440
Age																		
*Years*	1.01	1.00,1.01	0.0472	1.00	0.99,1.01	0.3804	1.01	1.00,1.02	0.0255	0.97	0.95,0.98	<0.0001	0.97	0.95,0.99	0.0021	0.97	0.95,0.98	0.0004
Mental health symptom score																		
Normal (reference)																		
Mild	0.89	0.68,1.16	0.3822	0.82	0.56,1.20	0.3095	0.94	0.64,1.39	0.7747	1.25	0.74,2.12	0.4058	0.67	0.35,1.28	0.2251	1.66	0.83,3.33	0.1490
Moderate	1.36	0.95,1.95	0.0938	1.07	0.63,1.80	0.8003	1.83	1.12,3.00	0.0168	1.86	1.10,3.15	0.0205	1.02	0.51,2.03	0.9595	2.41	1.23,4.73	0.0102
Severe	0.59	0.38,0.92	0.0191	0.61	0.32,1.17	0.1360	0.64	0.35,1.17	0.1478	1.19	0.61,2.34	0.6083	1.16	0.42,3.25	0.7729	1.19	0.51,2.75	0.6864
Currently married																		
No (reference)																		
Yes	1.46	1.15,1.85	0.0019	1.93	1.37,2.72	0.0002	1.24	0.88,1.75	0.2259	1.60	1.07,2.40	0.0233	1.88	1.05,3.35	0.0328	1.53	0.88,2.66	0.1289
Religious																		
No (reference)																		
Yes	1.17	0.90,1.52	0.2357	1.01	0.68,1.50	0.9489	1.22	0.87,1.70	0.2472	1.86	1.17,2.96	0.0091	1.07	0.48,2.37	0.8712	2.28	1.27,4.10	0.0055
State																		
CA (reference)																		
LA	2.45	1.87,3.22	<0.0001	2.65	1.83,3.82	<0.0001	2.31	1.56,3.43	<0.0001	2.01	1.32,3.06	0.0011	4.64	2.25,9.60	<0.0001	1.44	0.83,2.50	0.1982

### Sensitivity analyses

Appendix Table 1 examines the association between experiences of IPV and first-time (relative to not first-time) firearm purchase. IPV victimization was not associated with first-time purchase in either unadjusted or adjusted models (Model 5 AOR 0.75, 95% CI 0.33–1.71, *p* = 0.5). However, IPV perpetration was associated with significantly greater odds of first-time firearm purchase in unadjusted models (Model 2 OR 2.89, 95% CI 1.24–6.73, *p* = 0.01) and marginally greater odds of first-time purchase in adjusted models (Model 5 AOR 2.49, 95% CI 0.92–6.73, *p* = 0.07).

Appendix Table 2 replicates Model 5 for each firearm outcome by state. Significant positive associations between past year IPV experiences and firearm ownership and purchase remain present at similar magnitudes in both states, with the exception of a slightly attenuated association between past year IPV victimization and firearm ownership in Louisiana (AOR 1.78, 95% CI 0.99–3.20, *p* = 0.052).

## Discussion

Study findings document a strong relationship between recent IPV and both firearm ownership and past year firearm purchasing. While findings are documented for both IPV victims and IPV perpetrators, we see particularly strong findings for IPV perpetrators, who are more than 5 times more likely than non-perpetrators to have purchased a new firearm in the past year. The odds of a new firearm purchase are more than 3 times greater for IPV victims than those who are not abused. These findings are alarming given evidence that the presence of a firearm in an abusive relationship increases the risk of fatal outcomes for the victimized partner, especially among black women in the U.S [13]. This relationship between IPV victimization and firearm purchasing may explain “the largest demographic increase we’ve seen” in firearm purchase among black American and other women during the pandemic.

Notably, the findings suggest little difference between men and women with regards to IPV experience and firearm purchase or ownership. Relationship abuse appears to be associated with a similar increase in the propensity that men and women purchase or own a firearm. While many victims, particularly women, seek out weapons for self-protection, existing literature suggests these victims are rarely safer after becoming firearm owners.

The costs of the IPV-firearm relationship are too high to ignore. Firearm-IPV holds long-term implications for victims, who experience increased rates of stress, trauma, post-traumatic stress disorder (PTSD), depression, and other mental health issues. This violence also has physical manifestations, including chronic pain, migraines, and sleep disturbances [25–27]. More dire is the number of women killed by intimate partners in America. More than 6,000 Americans were murdered by their spouses in 2021 alone, and most of these fatalities were women [28].

A recent estimate suggests fatal firearm-related IPV costs California nearly $1.2 billion [29], while it costs Louisianans $1.6 billion [29]. Many of these costs are being driven by criminal justice expenditures. At the same time, women are being incarcerated at an increasing rate [30], with their crimes often resulting from IPV victimization [31, 32]. Although IPV victims are sometimes advised to obtain firearms to counter domestic violence threats, additional firearms can escalate the potential for harm and increase the potential for lethality, rather than offering true protection [4, 5]. Mukamal et al. 2024 found that 82% of California women incarcerated for murdering their spouses faced domestic abuse in the year preceding their offenses [32]. About 75% of the women with manslaughter or murder criminal convictions were subjected to IPV in the lead up to their infractions, while more than 70% were deemed to be in extreme or severe danger from IPV [32]. Many of these women were first threatened by their partners with a firearm [32]. This type of “IPV-to-prison pipeline” [32], helps to explain the over-criminalization of black women in America, who face disproportionate rates of gender violence, and heightened interaction with the carceral state [33]. Women victims, especially women of color, are also deprived of the social support needed to escape this cycle [34]. These cumulative disadvantages should be considered when considering solutions to IPV-related crimes.

Findings should be viewed with recognition of certain study limitations. Cross-sectional data impede assessments of causality. We did not assess the temporality of IPV incidents and firearm purchasing, and are thus, unable to assert a causal relationship between these two variables. Further, our data structure precludes us from ruling out reverse causality; it remains possible and even likely that firearm acquisition drives intimate partner violence, which is the opposite of what we posit. This imprecision limits our ability to conclude that IPV spurs firearm purchasing. Reliance on self-report creates vulnerability to recall bias and social desirability bias; the latter would likely yield under-reporting of IPV, resulting in conservative estimates. Survey wording may encourage respondents to provide answers about the behaviors of other household members rather than, or in addition to their own individual behavior, which may lead to imprecision in the indicators. Two states limit generalizability of findings beyond these two states, but these states showing similar associations despite being very different in firearm climate and policy suggests that IPV as a risk for firearm purchase is unlikely a state-specific finding. These two surveys constitute the only such state-representative data examining IPV and firearm behavior, which impedes our ability to generalize beyond these cases. Limited number of gender diverse participants affects our ability to understand the potentially unique needs this population may face in terms of firearm-related IPV.

## Conclusion

These findings suggest that both IPV victimization and perpetration are associated with an increased risk of firearm purchasing and ownership. Importantly, we find no significant difference in the association between IPV and firearm acquisition between men and women; the odds of firearm purchasing/ownership are not different among men and women with recent IPV experiences. We also see no meaningful difference in findings by state in terms of the observed association, though we do see substantially higher firearm ownership and purchasing in Louisiana compared with California. This study contributes to the evidence suggesting IPV may encourage firearm purchasing behavior. Though our study design is unable to establish a causal connection between firearm ownership and IPV, our study shows an association between these factors, and does so for both women and men. To date, the literature on IPV victimization and firearm purchasing has largely centered the experiences of women. While this focus is reasonable given women’s heightened likelihood of IPV, an increasing number of studies draw attention to the relationship violence suffered by men [35].

Additional research is needed to help elucidate these findings. Particular attention should be paid to the role of reciprocal IPV in firearm purchasing and the escalation of violence. Our data cannot tell us whether IPV causes firearm acquisition (or vice versa), nor can they establish whether IPV was unidirectional, or whether self-reported perpetrators were also victimized. Many relationships are characterized by mutual violence [35]. Relatedly, although women tend to be disproportionately subject to IPV—specifically intimate terrorism [36] and fatal IPV [37]— they are not the only victims of such violence [35]. Raj et al. (2023) show that in California, individuals that identify as trans and non-binary experience the highest prevalence of IPV; more than three-quarters of non-binary adults have experienced IPV during their lifetimes [18]. Men also face IPV [35]. 38% of California men reported experiencing a form of intimate partner violence during their lifetimes. In Louisiana, 55% of women and 47% of men experience IPV at least once during their lifetimes. Studies also show that men tend to be more reluctant to seek help when facing abuse [38, 39], which begs the question of how men choose to cope with IPV, especially when they believe their masculinity is being assailed. The results of the present analysis offers suggestive evidence that men, who typically own and purchase firearms at higher rates, exhibit a similar likelihood of procuring weapons in the context of IPV as women. Future research should confirm whether this relationship is causal such that IPV victimization encourages both male and female survivors to seek out firearms for protection. If so, IPV among both men and women has the potential to exacerbate one of America’s largest public health crises, firearm violence. Finally, these effects exist in states with both restrictive and with lax firearm laws. Future research should examine whether these findings persist in a broader sample of states.

## Supplementary Information

Supplementary Material 1.

## Data Availability

The datasets generated and analyzed during the current study are available in the OpenICPSR repository, for California (https://www.openicpsr.org/openicpsr/project/199087/version/V1/view) and for Louisiana (https://www.openicpsr.org/openicpsr/project/199088/version/V1/view).

## Abbreviations

IPV: Intimate partner violence
CI: Confidence interval
AOR: Adjusted odds ratio
OR: Odds ratio
IPH: Intimate partner homicide
CalVEX: California violence experiences survey
LaVEX: Louisiana violence experiences survey
PTSD: Post-traumatic stress disorder PTSD
